# Using 3-Isocyanatopropyltrimethoxysilane to Decorate Graphene Oxide with Nano-Titanium Dioxide for Enhancing the Anti-Corrosion Properties of Epoxy Coating

**DOI:** 10.3390/polym12040837

**Published:** 2020-04-06

**Authors:** Weihang Li, Bojun Song, Shirui Zhang, Fan Zhang, Chang Liu, Nan Zhang, Huiling Yao, Yuanchang Shi

**Affiliations:** Key Laboratory for Liquid-Solid Structural Evolution and Processing of Materials (Ministry of Education), Shandong University, Jingshi Road 17923, Jinan 250061, China; jnwh19990909@163.com (W.L.); bj19941021@163.com (B.S.); 17853143187@163.com (S.Z.); zhangfan1176@163.com (F.Z.); LiuChang981021@163.com (C.L.); endiling@126.com (N.Z.); yaohuiling1982@126.com (H.Y.)

**Keywords:** graphene oxide, titanium dioxide, 3-isocyanatopropyltrimethoxysilane, epoxy, modification, anti-corrosion

## Abstract

In this paper, the graphene oxide loaded with nano titanium dioxide (TiO_2_–GO) was synthesized through 3-isocyanatopropyltrimethoxysilane (IPTMS) and characterized by Fourier transform infrared spectroscopy (FTIR), X-ray diffraction analysis (XRD), X-ray photoelectron spectroscopy (XPS), scanning electron microscopy (SEM), thermogravimetric analysis (TGA), and dispersion test. The results illustrated our modification was successful and TiO_2_–GO was transferred from hydrophilic to hydrophobic. That greatly enhanced the dispersity of TiO_2_–GO in epoxy through the observation of the coating morphology test. Moreover, the impact of TiO_2_–GO on anti-corrosion property in epoxy was investigated by Electrochemical Impedance Spectroscopy (EIS). Comparing to pristine particles including GO and TiO_2_, TiO_2_–GO could more significantly improve the resistance of corrosion with the help of IPTMS. Furthermore, the anti-corrosion mechanism of TiO_2_–GO in epoxy was tentatively proposed and discussed.

## 1. Introduction

Corrosion, as an unavoidable problem, occurs in the process of metal application, roughly leading to the direct and indirect economic loss of several hundred billion every year [1]. In reaction to this phenomenon, protective coatings are often conducted on the metal substrates to cut the loss as much as possible. Because of the unique advantages, including the low shrinkage rate, the relatively low cost, the high bonding strength, and the outstanding corrosion resistance, etc [2,3,4], epoxy becomes one of the most frequently used coatings among the organic coatings. However, epoxy also has its own flaws that limit its further application like high brittleness, low tenacity and thermal shock resistance, and poor friction and wear properties [5]. Moreover, another issue is that the film would generate micro-pores with the solvent evaporating during the solidification. That would result in the more severe penetration of all gases and salts such as O_2_, H_2_O, and Cl^−^ [6,7]. To overcome this shortcoming, inorganic fillers like carbon black, clay, and silica were often added to the films, especially in practical use [8,9,10]. According to Liu et al., with the doping of the functional talcum powder, the corrosion performance of the resin enhanced enormously [11]. Ahmed et al. [12] prepared core-shell ferrites/kaolin pigments that combine the properties of both and exhibit better corrosion protection properties. While, recently, some experts attempted to incorporate the nano-scale fillers to the coating and found that it could enhance the property of coating more effectively [13,14,15]. Palraj et al. [16] compared and evaluated the performance of nano-silica and micro-silica epoxy composite coatings with the methods of salt spray test and EIS test, whose experimental results reflected that epoxy with nano-silica had better barrier properties. Ramezanzadeh and Attar [17] explored the size effect of ZnO particles on the corrosion resistance behavior in epoxy-polyamide coating. It was found that nano-scale ZnO could make more contribution to improving the anti-corrosion performance than the micro sized pigments.

Graphene oxide (GO), as a new century 2D nanomaterial, has attracted much attention in the last few years [18]. Due to the exceptional capabilities of great electrical [19] and thermal conductivity [20], high rigidity [21], high specific surface area, and optical transparency [22], GO has been widely explored for the applications in sensors [23], catalysis [24], energy conversion and storage [25], etc. In respect of coating, GO is an excellent filler, for it could improve not only the mechanical but also antiseptic properties [26,27,28]. However, based on GO’s characteristics of natural agglomeration and the difference of the applicable system, there is a problem of compatibility between organic matrix and GO. Therefore, GO usually needs to be modified before using since the properties of composites depend heavily on the dispersion of GO in the system and the interface interaction between them. One of the most common approaches is to grafting other nano hydrophobic particles on it. The advantage of this method is that it could increase the layer spacing, making GO turn into the loose state as well as preserve the properties of GO [29,30]. Furthermore, the grafted nano-particle would also enhance the coating performance because of its characteristics. There are many nano-particles suitable for decoration, such as SiO_2_, ZnO, Al_2_O_3_, and TiO_2_. Many experts have done a lot of related researches, while only a few are associated with GO decorated by nano-titanium dioxide (nano-TiO_2_). However, according to the existing results, nano-TiO_2_ was proved to have a positive impact on corrosion resistance of organic coatings [14,31,32]. 

**Figure 1:**
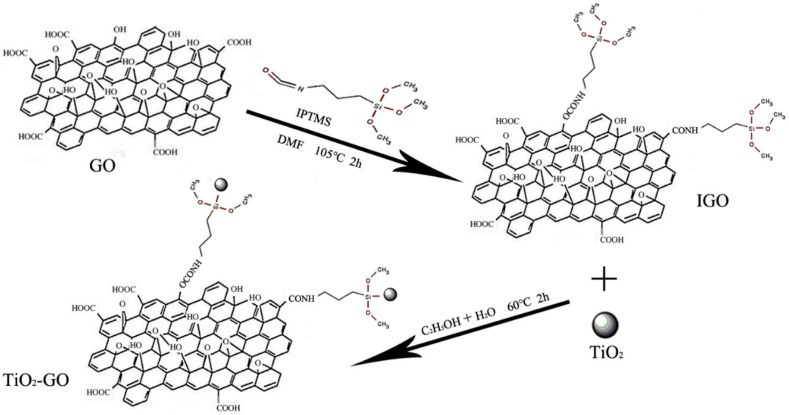
The mechanism of modification of graphene oxide (GO).

Therefore, in this paper, we put forward a new approach to modify GO with nano-TiO_2_ by 3-isocyanatopropyltrimethoxysilane (IPTMS), and the composition, structure, and morphology of TiO_2_–GO were characterized. The TiO_2_-GO nanocomposites were synthesized, utilizing IPTMS in this work. The specific reaction process is shown in Figure 1. Isocyanate group-terminated (–N=C=O), on the one side of IPTMS, reacted with hydroxyl (-OH) and carboxyl (–COOH) of GO. The methoxy group (–OCH_3_), on the other side, conjugated to the nano-TiO_2_ via the dehydration condensation reaction. Considering the high reactivity of IPTMS, our method is much easier to realize, and it helps to improve the reaction efficiency. In addition, the GO, nano-TiO_2_, and TiO_2_–GO nanocomposites were added into epoxy, respectively, with a low weight loading. Then, the corrosion resistance behavior and dispersion performance of all the films were tested and compared.

## 2. Experimental

### 2.1. Materials

The natural graphite powder (325 mesh) was provided from Beijing Jinglong special carbon graphene factory (Beijing, China). The epoxy resin and polyamide hardener (E44/651) were obtained from Phoenix Coatings Co., Ltd. (Wuxi, China). The nano-TiO_2_ and IPTMS were supplied by Shanghai Aladdin Biochemical Technology Co., Ltd. (Shanghai, China). The additives of coating (dispersant-2152, defoamer-085, leveling agent-320) were obtained from BYK-Chemie GmbH (Wesel, Germany). Ethanol (C_2_H_5_OH), acetone (CH_3_COCH_3_), butyl alcohol, xylene, N, N-dimethylformamide (DMF), sodium nitrate (NaNO_3_), 37% hydrochloric acid (HCl), 30% hydrogen peroxide (H_2_O_2_), 98% sulfuric acid (H_2_SO_4_), and potassium permanganate (KMnO_4_) were all purchased from Chinese medicine group chemical reagent co., Ltd. (Shanghai, China). The deionized water (DI) was self-made in the laboratory. All the reactants were used with no further purification. 

### 2.2. Preparation of TiO_2_–GO Nanocomposites

GO was synthesized by the Hummers method [33,34], and the preparation process of TiO_2_–GO nanocomposites went through two separate steps. The first step was preparing functionalized GO (F–GO) with the help of IPTMS. Another step was synthesizing TiO_2_–GO nanocomposites that decorating F–GO with nano-TiO_2_. The specific procedures were as follows: 

Firstly, 0.1 g GO was added in 50 mL DMF and sonicated for 20 min to be a homogeneous state. Then 1 g IPTMS was dropped in the solution under stirring at 105 °C for 2 h. Subsequently, the suspension was centrifuged and washed with anhydrous ethanol three times to remove the residual IPTMS and DMF to obtain F–GO. 

Next, the F–GO was dispersed in 25 mL ethanol and 0.03 g nano-TiO_2_ was dissolved in 25 mL DI both by ultrasound for 20 min. Subsequently, the nano-TiO_2_ was slowly dropped in the F–GO ethanol solution under rapid stirring at 60 °C for another 2 h. After that, the mixture was centrifuged and washed with DI and anhydrous ethanol three times, respectively. Lastly, the resultant product was dried at 55 °C for 24 h in the oven.

### 2.3. Preparation of TiO_2_–GO/Pure Epoxy (EP) Coating

In our experiment, firstly, 0.1 wt.% TiO_2_–GO, GO, and nano-TiO_2_ were separately dispersed in 10 g solvent under sonicating for 20 min. The solvent is the mixture of butyl alcohol and xylene in the ratio of 3:7 wt. Then 30 g epoxy was mixed in the suspension, using a glass rod to stir it until the resin dissolved in the solvent. Next, adding corresponding quantity hardener and coating additives (dispersant-2152, defoamer-085, leveling agent-320), the quantity ratio of resin and hardener is 3:1, kept stirring to let resin and hardener mix homogeneously. Making the bubbles rise out of the surface by setting down for 10 min, the TiO_2_–GO/EP coating was finished. 

Finally, the prepared coating would be applied on the treated tinplate panels with a dimension of 120 by 50 by 0.28 mm. Then before brushing, the panels were all abraded by 400, 800, and 1200 grades of sandpapers and washed by DI and acetone to remove oil and impurities. For the performance comparison, four sets of samples were conducted and cured at room temperatures for 120 h; they are pure EP, GO/EP, TiO_2_/EP, and TiO_2_–GO/EP. The final thickness of the coating layer was about 100 ± 5 μm for the corrosion test.

### 2.4. Characterization

#### 2.4.1. TiO_2_–GO Nano-Particles Characterizations

Spectroscopy analyses of the TiO_2_–GO nano-particles were measured by FT-IR and XPS. The FT-IR spectrums were recorded by using a Bruker Vector-22 infrared spectrometer (Bruker, Karlsruhe, Germany) over the wave number range of 400–4000 cm^−1^. The testing samples were prepared through the potassium bromide pellet method. To determine the composition, XPS experiments were employed by using a Thermo Scientific Escalab 250Xi spectrometer (Escalab, Gillingham, UK) that equipped with AI K_α_ X-ray source. The shift of binding energy was calibrated which was based on the C1s peak (285 eV). The phase crystal structures of GO, nano-TiO_2_, and TiO_2_–GO were examined by X-ray diffraction analysis via using Bruker D8 Advance (Bruker, Karlsruhe, Germany). The diffraction pattern was collected at a scan rate of 5°/min in the range of 5–90°. The morphology of GO and TiO_2_–GO were obtained through scanning electron microscopy (SEM). By using SEM JEOL-6701F (JEOL, Saitama, Japan), the microstructure of nano-particles was observed. Thermo-gravimetric analysis (TGA) of the particles was tested by model Mettler Toledo (Mettler Toledo, Manchester, UK) with a heating rate of 10 °C/min under nitrogen atmosphere, the temperature region is from 30 to 700 °C. The dispersion tests were also carried out to examine whether the nano-particles was hydrophilic or hydrophobic. In that section, 15 mg GO and TiO_2_–GO were added into 15 mL water and ethanol in a sample bottle separately, four samples were sonicated for 20 min, observed, and recorded the phenomenon after setting 1 h and 3 h, respectively. 

#### 2.4.2. Test of TiO_2_–GO /EP Coatings

Two kinds of tests were carried out in this section. One was to observe the state of TiO_2_–GO in EP by SEM. The other was to investigate and compare the anti-corrosion property of four samples via the EIS test by using Zahner_IM6e (Zahner, Saxony, Germany). The test system consisted of a three-electrode cell including a saturated calomel electrode, a platinum electrode, and a coated tinplate were used as reference, counter and working electrodes. The measure of the working electrode area that revealed to 3.5% NaCl solution was 3.799 cm^2^. Our measurements were recorded after immersing for 1 h and 120 h, respectively. The amplitude sinusoidal voltage in test was set as 10 mV with the frequency range from 10^−2^ Hz to 10^5^ Hz.

## 3. Results and Discussion

### 3.1. FT-IR Spectroscopy

**Figure 2:**
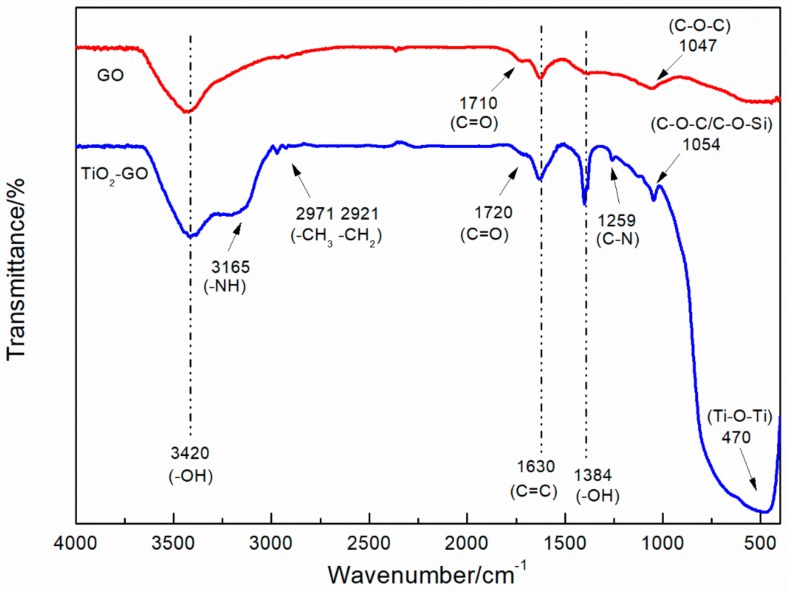
FT-IR spectra of TiO_2_–GO and GO.

FT-IR test was performed to investigate the functional group of GO and TiO_2_–GO particles. The FT-IR spectra of the samples is shown in Figure 2. For GO, the characteristic absorption peaks at 3420 cm^−1^ nearby indicate the hydroxyl groups. The rest peaks of GO include C–O vibrations of epoxide at 1047 cm^−1^, C=O and O–H vibrations of carboxyl groups at 1710 and 1384 cm^−1^, and C=C skeletal vibrations at 1630 cm^−1^ [28,35]. Regarding the spectra of TiO_2_–GO, it not only remains all characteristic absorption peaks of GO but also owns its particular peaks from IPTMS including the –NH and C–N stretching vibration of secondary amines at 3165 and 1259 cm^−1^, symmetric and asymmetric stretching vibrations of –CH_3_ and –CH_2_ groups at 2971 and 2921 cm^−1^ [36,37,38]. In addition, a broad and intense peak from nano-TiO_2_ that represents Ti–O–Ti could be seen at the low wavenumber region. At 1054 cm^−1^ nearby, the C–O vibrations peak becomes more intense and sharper, that is attributed to the overlap with the bending vibration of C–O–Si peak [39]. According to that peak, it could be concluded that GO has reacted with IPTMS molecules and the reaction products contains Ti–O–Ti bonds.

### 3.2. XRD Analysis

**Figure 3:**
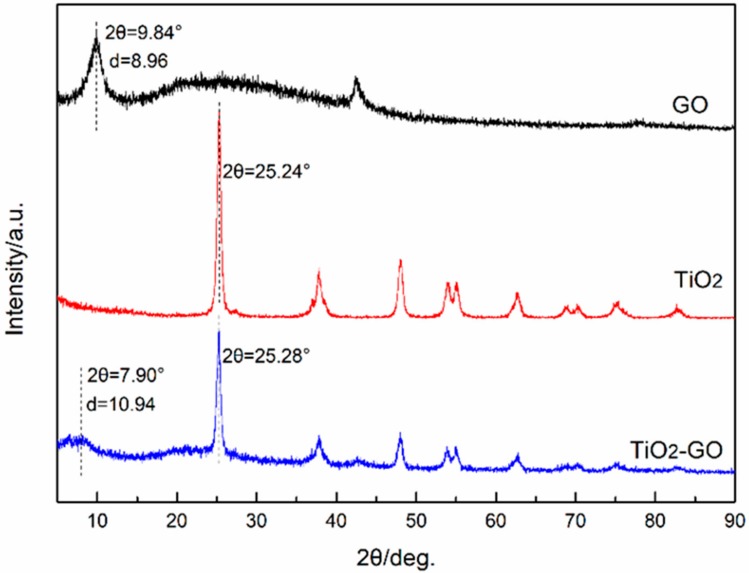
XRD patterns of GO, nano-TiO_2_, and TiO_2_–GO.

The XRD patterns which are about GO, nano-TiO_2_, and TiO_2_–GO are presented in Figure 3. It could be observed that TiO_2_–GO possesses both the characteristic diffraction peaks of GO and nano-TiO_2_, and the corresponding peaks are observed at 9.84° for GO and 7.90° for TiO_2_–GO. According to Bragg’s law:

nλ = 2dsinθ(1)

the D-spacing of our particles could be obtained by calculating. Therefore, there is an increment of D-spacing from 8.96 Å to 10.94 Å. That may attribute to the process of decoration; the closely-stacked structure of GO turns to be loosened during the reaction. On the other hand, this indicates the impact of nano-TiO_2_ on the GO. 

### 3.3. XPS Analysis 

**Figure 4:**
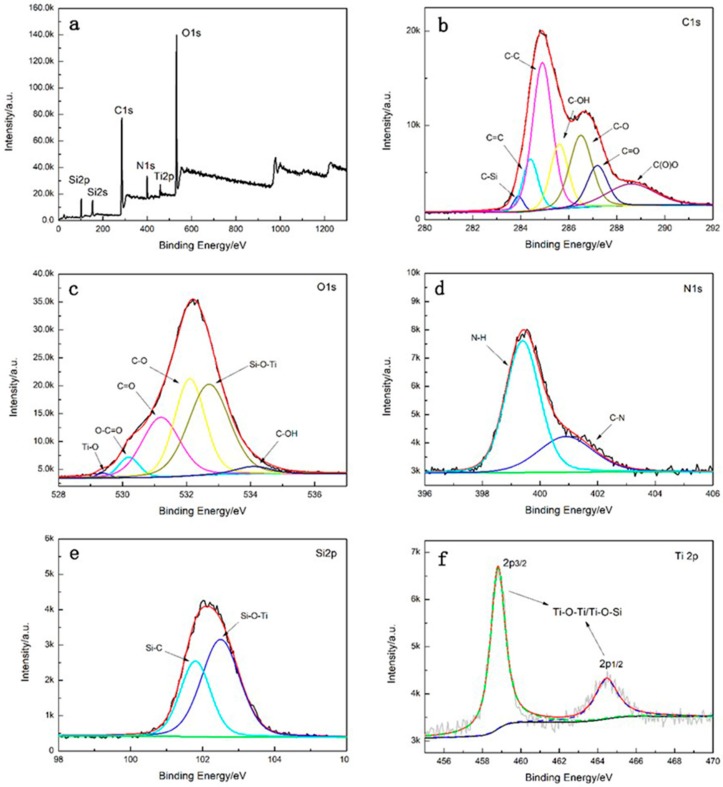
XPS survey spectra (**a**) and high resolution of spectra of (**b**) C1s, (**c**) O1s, (**d**) N1s, (**e**) Si2p, and (**f**) Ti2p of TiO_2_–GO.

XPS test was performed to examine the exact element that composed of TiO_2_–GO as well as the chemical bonds [40]. The more detailed information is exhibited in Figure 4. The XPS spectrum indicates that the corresponding peaks including C 1s, O 1s, Ti 2p, N 1s, Si 2s, and Si 2p suggest TiO_2_–GO is composed of these elements. According to the high resolution spectra, the C 1s are decomposed into several peaks including C–Si (283.9 eV), C=C (284.4 eV), C–C (284.9 eV), C–OH/C–O–Si (285.6 eV), C–O (286.5 eV), C=O (287.2 eV), and C(O)O (288.6 eV). The O 1s peak is divided into six Gaussian-Lorentzian peaks of O–C=O (530.2 eV), Ti–O (529.4 eV), C=O (531.2 eV), Si–O–Ti (532.7 eV), C–O (532.1 eV), and C–OH (534.1 eV). The N 1s spectrum is fitted in N–H (399.4 eV) and N–C (400.9 eV). Additionally, the Si 2p is separated into Si–C and Si–O–Ti whose binding energy is 101.8 eV and 102.5 eV, respectively. The Ti 2p is including Ti–O–Ti/Ti–O–Si at 458.8 eV and 464.5 eV [30]. The existence of Si–O–Ti, N–C, C–Si, N–H, and Ti–O–Ti peaks suggest that GO is modified with nano-TiO_2_ by IPTMS.

### 3.4. SEM Morphology

**Figure 5:**
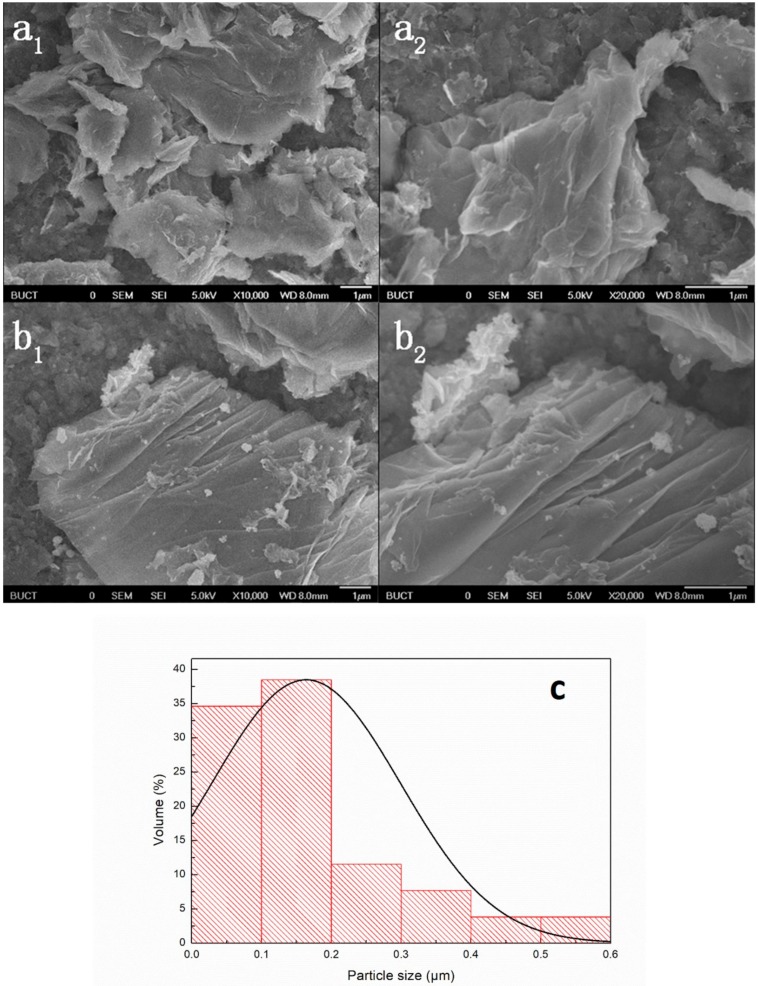
SEM image (**a_1_**,**a_2_**) GO, (**b_1_**,**b_2_**) TiO_2_–GO, and (**c**) The particle size distribution of b_1_.

Figure 5 describes the morphology of GO and TiO_2_–GO. Under this method, we can visualize the existence of nano-TiO_2_ on the GO. The particle size distribution of Figure 5b_1_ is shown in Figure 5c. It could be calculated that the average diameter of the nano-TiO_2_ is about 160 nm. By comparison of Figure 5a_1_,a_2_,b_1_,b_2_, it is evident that TiO_2_–GO turns to be unfolded and loose after the modification. This phenomenon is consistent with the result of XRD that the D-spacing is indeed increasing. However, the pristine GO seems to be aggregated to a certain degree. It may be related to the way of ordinary drying rather than the freeze-drying. Both of them show the characteristic wavy wrinkles on their surface. It could prove that our preparation and modification are successful and the observation of nano-TiO_2_ on the GO surface would also illustrate their connection is by covalent bonds rather than the physical deposition. 

### 3.5. TG Analysis 

**Figure 6:**
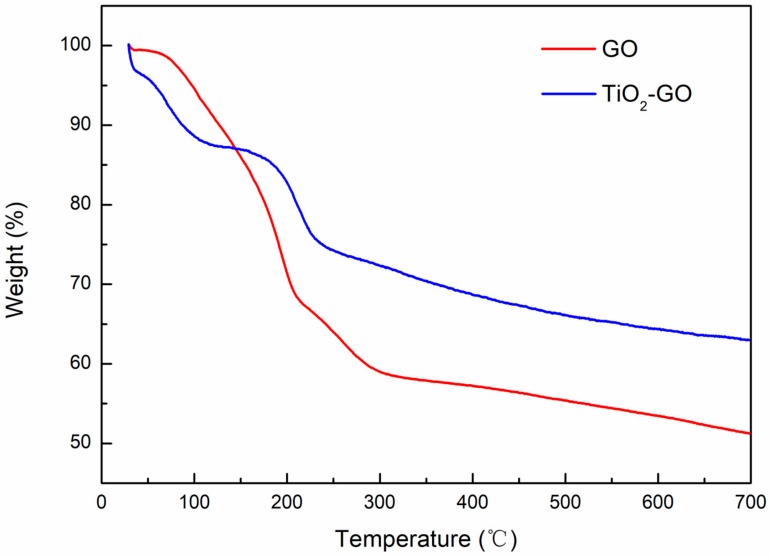
TGA curves of TiO_2_–GO and GO.

Figure 6 shows more details about the TGA thermograms of GO and TiO_2_–GO. From starting temperature to 120 °C, TiO_2_–GO suffered a small weight loss (12 wt.%). That is on account of the evaporation of the crystal water that forms in the process of synthesis [41]. At around 220 °C, the remained oxygen functional groups began to disintegrate which led to another loss (25 wt.%). The same phenomenon also occurs for GO. However, its mass loss is nearly 35 wt.%, which is much more than TiO_2_–GO. That is attributed to the more account of the functional groups on the surface of GO. At a higher temperature region, GO’s framework would be decomposed [42]. As a sort of inorganic particle, TiO_2_ has excellent heat resistance with a high melting-point. Therefore, TiO_2_–GO exhibits better thermal stability comparing to GO. The total mass loss is about 37 wt.% for TiO_2_–GO and 49 wt.% for GO. That further demonstrates the existence of nano-TiO_2_.

### 3.6. Dispersion Test

**Figure 7:**
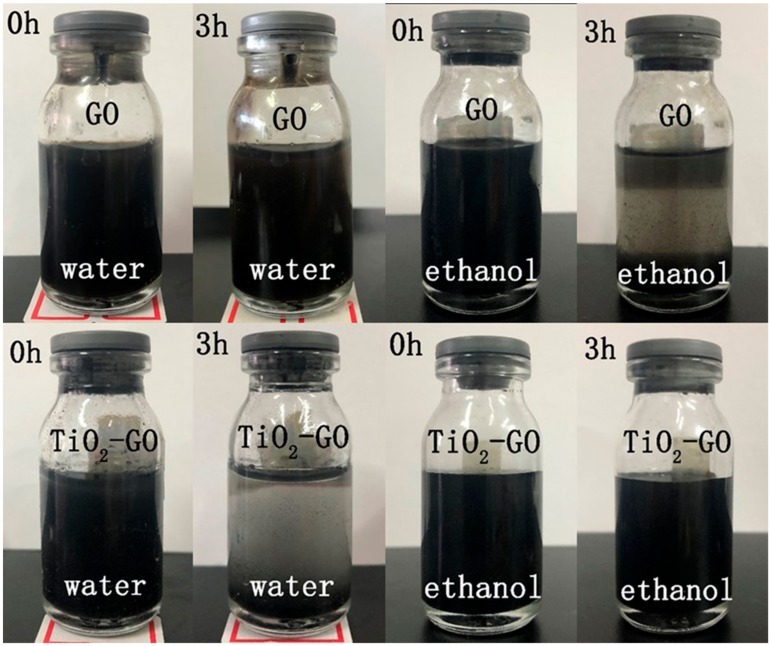
The comparison of TiO_2_–GO and GO suspension in water and ethanol after setting for 1 h and 3 h.

Figure 7 exhibits the dispersity of GO and TiO_2_–GO in water and ethanol at a concentration of 1 mg/mL. After sonification, the color of the two systems are a little different. The GO solution is dark brown, nearly black, while the TiO_2_–GO is gray-black. After the modification, the character of TiO_2_–GO changed from hydrophilic to hydrophobic. It precipitated in the water while GO remained well-dispersed after 3 h. However, TiO_2_–GO could be homogeneous in ethanol while GO could not maintain a stable state. That mainly attributes to the hydrophilic group –OH and –COOH react with IPTMS and decorated with nano-TiO_2_ at last. Both the nano-TiO_2_ and remaining epoxide groups are hydrophobic, so it could significantly improve its compatibility with the organic solvent. 

### 3.7. The Morphology of Epoxy Coatings

**Figure 8:**
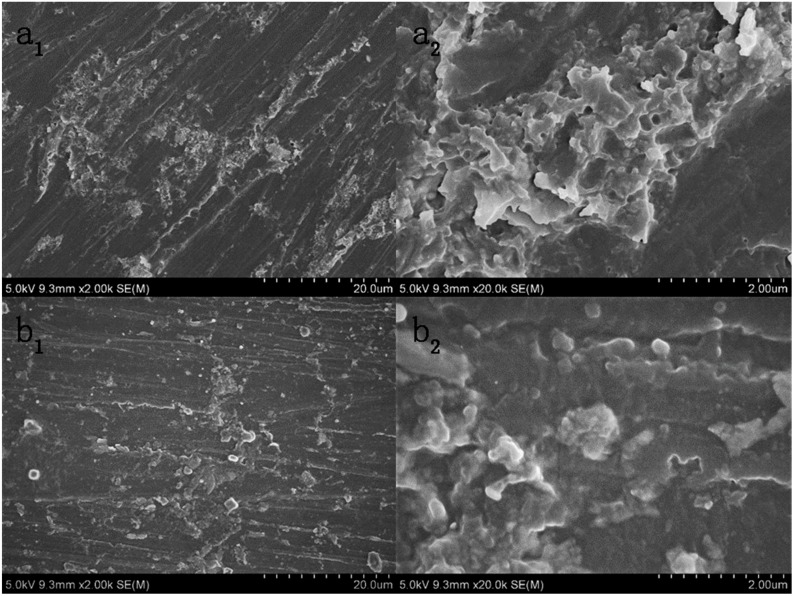
The morphologies of GO/EP (**a_1_**,**a_2_**) and TiO_2_–GO/EP (**b_1_**,**b_2_**).

Figure 8 displays the morphologies of TiO_2_–GO/EP and GO/EP. It reveals the state of TiO_2_–GO and GO in EP. By contrast, with the help of IPTMS and nano-TiO_2_, TiO_2_–GO distributes more evenly in epoxy. From the high resolution of Figure 8a_2_, severe agglomeration of GO can be seen in the matrix. However, the agglomeration of TiO_2_–GO is much slighter. That is primarily ascribed to the difference of characteristics that TiO_2_–GO is hydrophobic while GO is hydrophilic. Figure 8b_2_ is a little vague due to the epoxy completely encapsulates the TiO_2_–GO and the dielectric characteristic of it. That could be further verified that the TiO_2_–GO possesses better compatibility with the epoxy, which is in accord with the above result.

### 3.8. EIS Measurement

**Figure 9:**
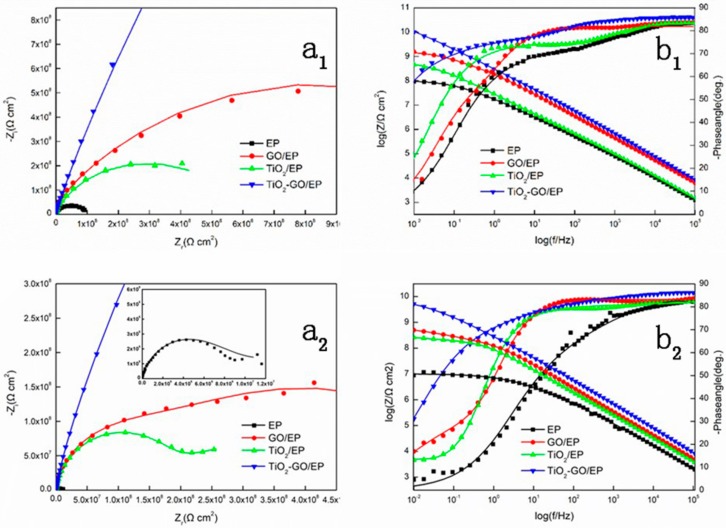
Nyquist(**a_1_**) and Bode(**b_1_**) spectrum after immersing 1 h; Nyquist(**a_2_**) and Bode(**b_2_**) spectrum after immersing 120 h.

For coated substrates, the process of corrosion happens in three steps: (1) the coated substrate fully contacts with the corrosive medium while it is not penetrated, (2) the corrosive medium diffuses in coating through the defects while not reaching the substrate, and (3) the corrosive medium reaches the metal surface and results in damage [27]. For better evaluation of its corrosion behavior, the EP, GO/EP, TiO_2_/EP, and TiO_2_–GO/EP was explored by EIS. This could be seen from Figure 9a_1_,b_1_, all samples display a semicircle shape in Nyquist diagrams while the diameter of TiO_2_–GO/EP is the largest among them. In general, the larger semicircle diameter is related to the lower corrosion rate [43]. Therefore, it means the corrosion rate of TiO_2_–GO/EP is the lowest. As the test proceeded, the result was also proved. The EP, GO/EP, and TiO_2_/EP appeared another semicircle after immersed 120 h which illustrated the electrolyte has penetrated in the coating while the film of TiO_2_–GO/EP is still in the stage of only one semicircle. That means TiO_2_–GO plays an important role in epoxy for anti-corrosion, it distributes uniform in the system that making the film much denser to impede the electrolyte effectively.

Figure 9a_2_,b_2_ is the bode plots of four samples in different stages. The value of Z at low frequency (|Z|_0.01 Hz_) is a measure of corrosion resistance and higher |Z|_0.01_ Hz indicates a better corrosion resistant coating on the metal substrate [44]. All the films show a decent performance at the initiative phase especial the systems with nano-fillers. Furthermore, among three sorts of powders, the positive effect of TiO_2_–GO is the most significant; its value of |Z|_0.01 Hz_ reached 10^10^ Ω cm^2^ which was larger than the others. After immersion for about 120 h, all the film performance declined to varying degrees. The degradation of EP and GO/EP are more serious, especially the EP film, which is down by an order of magnitude from 10^8^ Ω cm^2^ to about 10^7^ Ω cm^2^. While the decline of nano-TiO_2_/EP and TiO_2_–GO/EP is not significant at the same time. That is primarily attributed to the excellent dispersity of the nano-filler. Therefore, it lays down in matrix homogeneously and forms a zigzag structure that extends the permeation distance of electrolyte. The change of phase angle of four samples could also reflect the barrier result. Only one relaxation time could be seen of all the films at the beginning stage. However, with the increment of the immersion time, all the samples appear two relaxation times except the TiO_2_–GO/EP, and its phase angle remains the largest, which is about −85°. That would illustrate the positive effects of nano-fillers on the film and the TiO_2_–GO/EP possesses the best anti-corrosion property.

**Figure 10:**
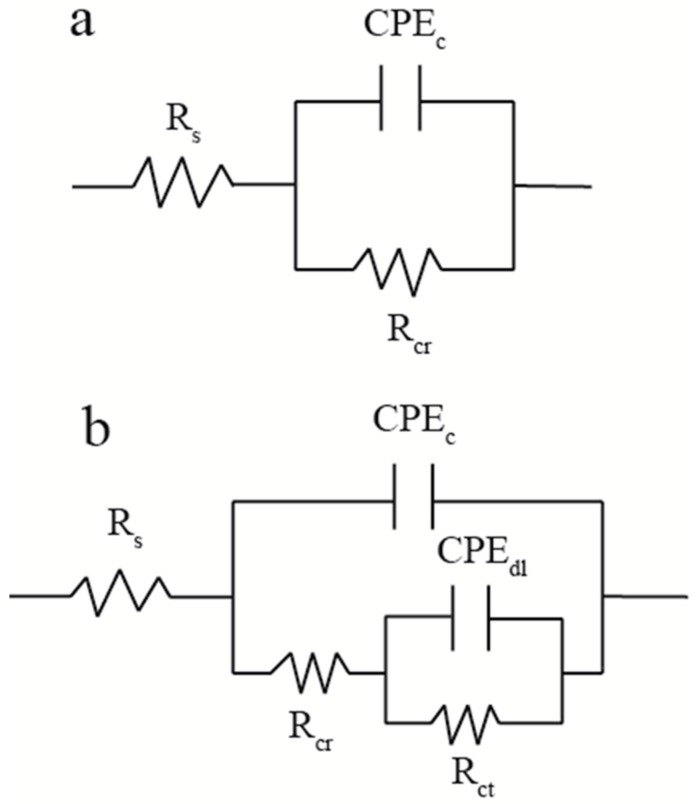
The equivalent electrical circuit used for fitting EIS data (**a**,**b**).

For better interpret the EIS data, the results are fitted by electrical circuit (Figure 10). The R_s_, R_cr_, R_ct_, CPE_c_, and CPE_dl_ represent the solution resistance, coating resistance, charge transfer resistance, and constant phase element of the coating and double layer, respectively. To model the electrochemical behavior of the system, a constant phase element associated with an exponent (0 ≤ n ≤ 1) was used to replace the capacitor for representing the experimental deviation from the semi-circle capacitance behavior since the heterogeneity of the coating surface [45]. The CPE could consider as a real resistance when n = 0 and a completely capacitance when n= 1. The fitting rules were as follows: The Nyquist diagram of the samples, which is one semicircle, was fitted by Figure 10a. In this period, a small amount of electrolyte has diffused in the coating which leads to the R_cr_ decreases with the immersion time increases. Therefore, the R_ct_ is no longer considered infinite, that could not be ignored in fitting process. The rest with two semicircle samples were fitted by Figure 10b. At this stage, the electrolyte reaches the substrates and reacts with it. That results in the lower value of R_ct_ which need to be fitted in parallel with CPE_dl_. The electrochemical parameters after fitting are shown in Table 1.

## 4. Conclusions

In this paper, we put forward a novel technique method to decorate GO with nano-TiO_2_ by using IPTMS. One of the advantages of IPTMS, comparing to the regular silane coupling agent like KH550 or KH560, is that it could react with all the hydrophilic groups of GO (–OH and –COOH) and preserve the hydrophobic group (–CH(O)CH–). Therefore, after the modification, TiO_2_–GO would possess better compatibility with epoxy in theory, which is a crucial factor in improving the performance. Through the characterization results, it can be obtained that with the help of IPTMS nano-TiO_2_ successfully combined with GO through chemical bonds, and it indeed exhibits the good dispersity in the solvent system. In the meantime, the TiO_2_–GO/EP shows the outstanding anti-corrosion property from the EIS test. The results confirm that according to our modification methods, the combination of GO and nano-TiO_2_ could achieve the effect that one plus one is greater than two. Furthermore, these methods could be not only applied in this field, but also the other relevant area.

## Figures and Tables

**Table 1:** The electrochemical parameters after fitting of all samples immersed in 3.5% NaCl solution for 1 and 120 h.

Sample	Time/h	R_s_(Ω cm^2^)	Q_c_		R_cr_(Ω cm^2^)	Q_dl_		R_ct_(Ω cm^2^)
			Y_0_(Ω^−1^ cm^−2^ s^n^)	n		Y_0_(Ω^−1^ cm^−2^ s^n^)	n	
EP	1 h	0.01	6.766 × 10^−8^	0.8895	5.64 × 10^7^			
GO/EP	1 h	0.01	9.365 ×10^−9^	0.894	1.173 × 10^9^			
TiO_2_/EP	1 h	0.01	5.854 × 10^−9^	0.8639	4.939 × 10^9^			
TiO_2_−GO/EP	1 h	0.01	5.869 × 10^−10^	0.9014	1.501 × 10^10^			
EP	120 h	0.01	2.702 × 10^−9^	0.9102	9.947 × 10^5^	2.572 × 10^−11^	0.4752	1.098 × 10^7^
GO/EP	120 h	0.01	3.119 × 10^−9^	0.9243	7.939 × 10^8^	4.92 × 10^−9^	0.7169	5.988 × 10^8^
TiO_2_/EP	120 h	0.01	9.607 × 10^−9^	0.9218	1.625 × 10^8^	6.08 × 10^−9^	0.5517	5.44 × 10^8^
TiO_2_−GO/EP	120 h	0.01094	6.24 × 10^−10^	0.8986	5.149 × 10^9^			
